# HSV-1 as a Potential Driver of Alzheimer’s Disease

**DOI:** 10.3390/pathogens14101022

**Published:** 2025-10-08

**Authors:** Dar-Yin Li, Eun Seok Choi, Xiaoyong Bao

**Affiliations:** 1 John Sealy School of Medicine, The University of Texas Medical Branch, Galveston, TX 77555, USA; darli@utmb.edu; 2 Department of Pediatrics, The University of Texas Medical Branch, Galveston, TX 77555, USA; eunchoi@utmb.edu; 3 The Institute of Translational Sciences, The University of Texas Medical Branch, Galveston, TX 77555, USA; 4 The Institute of Human Infections and Immunity, The University of Texas Medical Branch, Galveston, TX 77555, USA

**Keywords:** HSV-1, Alzheimer’s disease, dementia pathogenesis, HSV-1 experimental models

## Abstract

Herpes simplex virus type 1 (HSV-1) is a continuous health challenge, and current antiviral treatments cannot cure the virus. As life expectancy continues to increase worldwide, HSV-1 should remain a focus to minimize its associated health complications within the aging population. While often asymptomatic, HSV-1 causes oral and cutaneous lesions and establishes latency with periodic reactivation. Antivirals reduce symptoms but do not eradicate the virus. Emerging evidence links HSV-1 to Alzheimer’s disease (AD) via chronic neuroinflammation, amyloid-beta and tau accumulation, oxidative stress, and synaptic dysfunction, with viral proteins detected in AD-affected brain regions. This review assesses the current evidence for HSV-1 in dementia pathogenesis, examines antiviral strategies as potential neuroprotective interventions, and outlines the experimental models required to establish causality.

## 1. Introduction

Globally, approximately 4 billion people, or 64% of the population under the age of 50, are infected with herpes simplex virus type 1 (HSV-1), making it a significant public health concern due to its lifelong and incurable nature [1]. HSV-1 is a linear, double-stranded DNA virus, classified under the Simplexvirus Genus, Alphaherpesviridae Subfamily, and Herpesviridae Family. It is primarily transmitted through contaminated saliva or other bodily secretions that are infected [2]. While often asymptomatic, it can cause oral lesions, herpetic whitlow, gingivostomatitis, or vesicles on an erythematous base, and, in some cases, is linked to neurodegenerative diseases [2,3]. Current clinical diagnostic tools for HSV-1 consist of HSV-1 serology, viral polymerase chain reaction (PCR), Tzanck smear, viral culture, and direct fluorescence antibody assay [2].

Antiviral medications such as acyclovir, famciclovir, and valacyclovir are prescribed to symptomatic patients. A complete cure for HSV-1 remains elusive in 2025, as these medicines do not eliminate the virus. After an initial infection, HSV-1 often enters a latent state, which can be reactivated, causing recurrent outbreaks, symptomatic or asymptomatic [4]. Emerging evidence suggests that HSV-1 may contribute to neurodegeneration, particularly in AD, potentially through mechanisms such as chronic neuroinflammation, amyloid-beta (Aβ) and hyperphosphorylated Tau accumulation, oxidative stress, and synaptic dysfunction [5]. Moreover, HSV-1 proteins have been detected in the hippocampus and thalamus, both of which are affected in AD. However, the role of HSV-1 in dementia remains unclear. In this review, we examine current evidence on the potential role of HSV-1 in the pathogenesis of dementia and consider whether targeting HSV-1 could be a viable strategy for preventing progressive neurodegeneration. We also discuss experimental models needed to advance research in this area.

## 2. Epidemiology

### 2.1. Medical Burden of HSV-1

In 2020, approximately two-thirds of the global population under the age of 50, about 4 billion people (64%), harbored HSV-1 infection, including approximately 376.2 million individuals aged 15–49 experiencing genital HSV-1 infection, the highest prevalence reported in the Americas [6]. Although overall HSV-1 prevalence in the United States has declined over time, it still varies markedly by race: in 2015–2016, age-adjusted oral and genital HSV-1 prevalence was highest at 71.7% among Mexican-American individuals and lowest at 36.9% among non-Hispanic whites [7].

A United Kingdom (UK)-based cross-sectional study found that 63.6% individuals are HSV-1 seropositive, and the prevalence of frequent HSV-1 reactivation is 17.6% of individuals aged 40–79. Risk factors significantly associated with HSV-1 seropositivity included lower socioeconomic status, having four or more siblings, and living in an urban environment. In contrast, frequent reactivation was primarily linked to kidney disease along with fatigue, stress, and outdoor occupational exposure; no strong associations with other clinical comorbidities were observed [8].

Moreover, data from U.S. population surveys indicate that current smokers have a significantly higher risk of HSV-1 infection, and risk increases with higher levels of nicotine exposure, as measured by serum cotinine [9,10].

### 2.2. Alzheimer’s Disease and Other Dementias

According to the World Health Organization, about 57 million people have dementia worldwide, which costs about $1.3 trillion. Women are more likely to be affected by dementia than men as of 2019 [11]. In the United States, dementia affects 6 million Americans and contributes to more than 100,000 deaths per year, and an estimated lifetime risk of acquiring dementia is 42% with higher risk in women, the African American population, and APOE e4 carriers, ranging from 45% to 60% [12,13]. Among dementias, AD is the most common type of dementia, and abundant evidence shows that there is an association between dementia and HSV-1 [14,15,16,17]. In this subsection, we will briefly describe non-AD dementia, while AD and the evidence of HSV-1 as a risk factor of AD will be discussed in a separate section.

#### 2.2.1. Frontotemporal Dementia

Frontotemporal dementia, also known as Pick’s disease, involves atrophy of the frontal and temporal lobes. A majority of individuals inherit autosomal dominant mutations of C9orf72 on chromosome 9, progranulin on chromosome 17, and microtubule-associated protein tau on chromosome 17 [18]. Individuals are usually younger than age 65, presenting with an early onset of personality and behavioral changes, and progression of aphasia along with some movement disorders [19]. Hyperphosphorylated tau and aggregation of TAR DNA-binding protein 43 (TDP-43) are seen in histology of the frontal and temporal lobes, leading to neurodegeneration [20]. Currently, minimal studies have been conducted to examine the association between HSV-1 and frontotemporal lobe dementia. One study showed an adjusted risk ratio of 1.778 (CI 0.726, 4.352), but it did not reach statistical significance [21].

#### 2.2.2. Lewy Body Dementia

Lewy Body dementia (LBD) is usually not directly inherited in the way some single-gene disorders are, most cases are sporadic, but rare familial cases exist as well. Familial LBD cases often linked to mutations in genes such as synuclein-α (SNCA), glucocerebrosidase (GBA), or others associated with Parkinson’s disease [22]. Individuals present with visual hallucinations, fluctuating cognitive alertness, sleep disorders, and Parkinsonism [23]. Lewy bodies are intracellular eosinophilic inclusions made of α-synuclein proteins that contain neuromelanin, primarily found in neurons of the brainstem, limbic system, and cerebral cortex [23]. The association between HSV-1 and Lewy Body dementia remains to be explored, with an adjusted risk ratio of 1.398 (CI 1.084, 1.804), but without statistical significance [21].

#### 2.2.3. Vascular Dementia

Vascular dementia is a multifactorial disease that is often caused by multiple arterial infarcts and chronic ischemia. The infarct usually presents with a stepwise decline in cognitive function and late-onset memory impairment, with focal neurological deficits depending on where the occlusion(s) occurred [24]. Because vascular dementia is from an infarct or ischemia, exploring HSV-1 with vascular dementia is difficult and requires an examination of how HSV-1 impacts older adults’ vascular system, which is beyond the scope of this review.

## 3. HSV-1 Pathogenesis

### Transmission, Infection, Latency, and Reactivation

HSV-1 is transmitted through saliva, contaminated bodily fluids, or skin-to-skin contact with lesions, such as fluid-filled blisters and crusts, that contain the virus [2,25]. The incubation period from initial exposure ranges from one to 26 days, and HSV-1 shedding occurs only from specific body regions [25]. While most immunocompetent individuals remain asymptomatic and experience self-limiting infections, immunocompromised patients may develop disseminated diseases, such as herpes esophagitis, aseptic meningitis, herpes encephalitis, or lymphocytic meningitis [26].

The virus primarily infects the epithelial cells of the oral and nasal mucosa, where lytic replication produces more viruses to enter the peripheral nervous system. HSV-1 usually use its glycoproteins gB and gC to attach to the receptor on the cell surface, heparan sulfate proteoglycans. Because heparan sulfate proteoglycans are ubiquitously expressed, many cell types can bind HSV-1. HSV-1 then use glycoproteins gD to interact with specific cellular receptors, including nectin-1, HVEM, or 3-o-sulfated hepatan sulfate to trigger conformational changes in viral glygoproteins, leading to membrane fusion, which always requires gH/gL and gB for the execution after gD-receptor binding [27]. HSV-1 utilizes retrograde axonal transport to facilitate its movement to the cell body of the sensory neurons [28]. Once HSV-1 reaches the sensory neurons and travels to the trigeminal ganglion, it establishes latency. Although latency predominantly resides in the trigeminal ganglion, HSV-1 is known to establish latency in the brain, possibly via the TG-brainstem connection, olfactory nerve, and the ocular routes [5,29,30]. During the latent period, the trimethylation of lysine 27 on histone H3 (H3K27me3) from the HSV-1 genome silences the lytic genes until a reactivating trigger activates a sub-population of the latent HSV-1 in the trigeminal ganglion [31,32]. Within neurons, HSV-1 in the cell body has the potential to become infectious viral particles, whereas axonal infection alone is ineffective unless the tegument protein, VP16, is added to the axons. Furthermore, low levels of VP16 and infected-cell protein 0 (ICP0), another tegument protein, potentially due to the long distance of the axons, result in reduced viral gene expression, which leads to latency in neurons [29,33,34]. HSV-1 latency establishment is also known to be sensitive to antiviral molecules such as IFN-λ and Toll-like receptors [35,36].

During reactivation, HSV-1 particles travel bidirectionally back to the sensory neurons and also to the brainstem, where they can travel farther to the thalamus, temporal lobe, and other brain regions in the central nervous system, resulting in neurological disorders and accumulation of intracellular damage from reactivated HSV-1 particles [5,29]. In the central nervous system, reactivated HSV-1 enters through the trigeminal and olfactory nerves. This causes encephalitis in the temporal lobe and the limbic system, and it subsequently spreads to other regions in the neocortex [37]. The trigeminal nerve, specifically the mandibular nerve, V3, innervates the lower face and jaw, where HSV-1 initial infection usually occurs; therefore, the trigeminal nerve is the predominant site for HSV-1 to enter the CNS. It has been shown that psychosocial stress, dental procedures, the common cold, and exposure to solar UV radiation increase the likelihood of HSV-1 reactivation [38,39,40]. Interestingly, an increase in immune-suppressive regulatory T cells is shown to suppress CD8+ T cells, leading to HSV-1 reactivation, while low-level regulatory T cells are seen in latent HSV-1 infection [41]. Recurrent HSV-1 infection in AD mice has been shown to accelerate the progression of Aβ and tau hyperphosphorylation in the dentate gyrus of the hippocampus, as well as the detection of HSV-1 in the neocortex and cerebellum [42,43].

## 4. Alzheimer’s Disease

### 4.1. Alzheimer’s Disease Inheritance

AD is the most common type of dementia and the leading cause of the neurodegenerative disease worldwide [11]. Other neurodegenerative diseases include Lewy body disease, Parkinson’s disease, Huntington’s disease, Amyotrophic lateral sclerosis, and Creutzfeldt-Jakob disease, along with reversible diseases such as thiamine deficiency from alcoholism and hypothyroidism.

While the etiology of AD is multifactorial, a combination of genetic susceptibility and environmental or social factors has been recognized to influence disease onset and progression. One of the most recognized genetic risk factors for late-onset AD (LOAD) among genetic contributors is the apolipoprotein E4 (ApoE4) allele, with approximately 12% of the general population carrying this variant [44,45]. Importantly, the relationship between ApoE4 and HSV-1 has drawn increasing attention, as HSV-1 infection is highly prevalent in older adults, and carriers of ApoE4 show increased vulnerability to infection-driven neurodegeneration [46]. In contrast, ApoE2 appears to be protective, being associated with a reduced risk of AD and even longevity [47]. This highlights how a single genetic factor may shape host–virus interactions and influence neurodegenerative outcomes.

By comparison, early-onset AD (EOAD) is less clearly connected to environmental triggers such as HSV-1, and our understanding remains limited. Most current knowledge is, therefore, confined to well-established genetic determinants, including mutations in amyloid precursor protein (APP) and presenilin genes (PSEN1, PSEN2), which drive excessive Aβ production [48]. Another related example is that individuals with Down syndrome (trisomy 21) are 40–80% more likely to develop AD characteristics by age 50 due to an increase in Aβ production resulting from an extra 21st chromosome, where APP is encoded [49]. Unlike LOAD, EOAD research relies heavily on canonical genetic factors, underscoring a significant gap in our understanding of whether and how viral infection may influence disease onset in younger individuals.

### 4.2. Alzheimer’s Disease Pathogenesis

Clinically, AD accounts for up to 80% of dementia cases and is characterized by memory loss, behavioral changes, and neurocognitive dysfunction with slow onset and steady decline [50]. Morphologically, AD brains exhibit widespread cortical atrophy, narrowing of the gyri, widening of the sulci, and enlargement of the central ventricles, resulting in hydrocephalus ex vacuo [51]. Histology of AD reveals Hirano bodies in the hippocampus, which are intracellular, eosinophilic proteinaceous rods, as well as neurofibrillary tangles and senile plaques in the gray matter [52].

The initial target of AD occurs in the hippocampus, often with the accumulation of Aβ produced from APP and neurofibrillary tangles. One of the most extensively studied hypotheses of pathogenesis is the Aβ hypothesis, which posits that Aβ accumulation triggers a cascade of kinases, including CDK5 and GSK-3, leading to tau phosphorylation [41,46,47]. Tau is a protein critical for microtubule stability in neurons. In AD, tau phosphorylation is disrupted due to abnormal post-translational modifications and hyperphosphorylation, leading to neurofibrillary tangles [50]. The increased neurofibrillary tangles promote the cytokine IL-1, an inflammatory marker, and prolong inflammation, resulting in sustained neuronal damage, as further discussed later [53]. Furthermore, tauopathy possesses similar behavior to prion proteins, where abnormal tau aggregates can propagate to healthy tau proteins, leading to neurodegeneration [54]. However, some individuals maintain normal cognition despite the presence of Aβ and tauopathy. In addition, the cause-and-effect relationship between Aβ, tau accumulation, and inflammation remains unclear [55,56].

## 5. The Association Between HSV-1 Infection and AD Development

AD is characterized by progressive memory impairment, executive dysfunction, and visuospatial impairment [57]. Several studies have shown that neurotropic viral infections serve as a risk factor for AD onset and progression. Herpesviruses, including HSV-1, human herpesvirus 6A and 7 (HHV-6A/7), cytomegalovirus (CMV), and other viruses such as varicella-zoster virus (VZV), human immunodeficiency virus (HIV), influenza, and severe acute respiratory syndrome coronavirus 2 (SARS-CoV-2), have all been reported to have the potential to increase AD-like changes in the brain [58,59,60,61,62,63]. Among those, HSV-1 has been most studied in AD. Regarding the contribution of HSV-1 infection to AD onset, the studies started with the observation demonstrating the association between HSV-1 DNA and plaques. 72% of HSV-1 DNA was associated with plaques, whereas only 24% of HSV-1 DNA was associated with plaques in normal brains [5,64]. Furthermore, HSV-1 DNA and proteins were found in the central nervous system, particularly in the hippocampus and thalamus, which are predominantly affected in AD, supporting the association between HSV-1 infection and AD [5]. In an epidemiological study, a meta-analysis revealed a positive correlation between anti-HSV-1 acyclovir treatment and the potential reduction in the risk of AD development or slowing down the progression of AD symptoms. However, the analysis may be limited by the lack of data from prospective randomized controlled clinical trials [65]. A Phase II randomized, double-blind, placebo-controlled trial of valacyclovir in patients with mild AD and evidence of HSV-1/2 infection was recently completed (Columbia University, NCT03282916). The study employed the modified ADAS-COG11 neuropsychological test to assess cognitive dysfunction in AD. After 78 weeks of treatment, valacyclovir did not slow disease progression. However, it remains unclear whether a longer treatment duration or intervention at an earlier disease stage might be required to observe therapeutic effects. Overall, the mechanisms underlying HSV-1 in regulating AD progression are unclear, and further experimental studies are needed to confirm the epidemiological association between HSV-1 and AD. In addition, it remains unclear whether the increased presence of HSV-1 DNA and proteins in brain regions is a consequence of AD-associated immune dysfunction, making the brain more susceptible to infection. In this section, we will discuss some potential mechanisms that support HSV-1 in the onset of AD.

### 5.1. Neuroinflammation

Neuroinflammation refers to the inflammatory response in the peripheral or central nervous system that occurs in response to an insult. Microglia, astrocytes, and oligodendrocytes function as scaffolds, tissue repair, neuronal growth, and synaptic remodeling in the central nervous system. When there is an accumulation of Aβ in AD brains, microglia function as macrophages, phagocytosing Aβ while also activating other inflammatory mediators and reactive oxygen species in the brain [66]. The prolonged neuroinflammation and increased cytokine production are shown to have reduced reuptake of Aβ, exacerbating the progression of AD with tau hyperphosphorylation and neuronal loss [67]. HSV-1 is known to induce microglia to produce cytokines and chemokines such as TNF-α, IL-1B, IP-10, and RANTES, while IL-10, an anti-inflammatory cytokine, can decrease inflammation caused by HSV-1 [66,68]. Therefore, recurrent episodes of HSV-1 reactivation may lead to prolonged microglia activation and chronic neuroinflammation, resulting in neuronal damage and increased levels of neurodegenerative markers, such as the accumulation of Aβ and tau. Although HSV-1 can infect microglia, it cannot complete its viral replication, but instead, infected microglia release various chemokines and cytokines, inducing neuroinflammation [69].

### 5.2. Oxidative Stress

Another hallmark of inflammation is the release of reactive species (ROS), mainly from mitochondria. In AD brains, dysfunction of the mitochondria inhibits energy metabolism and increases oxidative stress, resulting in neurodegeneration [70]. Specifically, Aβ aggregation disrupts ROS homeostasis in the brain, as these aggregates possess high-affinity binding sites for biometals such as iron, zinc, and copper. The increased concentration of copper acts as a mediator for the hydroxyl radical, inducing oxidative stress [70,71]. With that in mind, HSV-1 reactivation can also induce oxidative stress through the activation of IL-1 and TNF-α, inflammatory markers that activate microglia in the CNS and heighten inflammation [72]. Because reactivated HSV-1 infection independently activates microglia and increases ROS through the natural inflammatory response, this physiological behavior can exacerbate the abnormal mitochondria in AD neural cells, leading to greater neuronal loss and AD progression.

### 5.3. Other Inflammatory Mechanisms

Over the last decade, significant research has been conducted to explore the molecular changes and the mechanisms by which HSV-1 infection worsens AD. In one study, HSV-1 infection was found to activate NLRP3 inflammasome, which induces Aβ pathology in AD [73]. The NLRP3 inflammasome is part of the innate immune system, which is often activated by pathogen-associated molecular patterns (PAMPs) and damage-associated molecular patterns (DAMPs), leading to the downstream activation of caspase-1 that signals pro-inflammatory markers [74]. PAMP- and DAMP-triggered NLRP3 inflammasome then leads to neuroinflammation via IL-1β and IL-18 [75]. NLRP3 inflammasome also benefits the CNS by decreasing HSV-1 particles through microglia phagocytosis. However, this benefit is compromised by the inhibitory impact of HSV-1 on Golgi apparatus fragmentation and Golgi matrix protein 130, which downregulate TLR3 and reduce the microglial response towards HSV-1, enabling HSV-1 to escape phagocytosis while continuing to induce NLRP3 [76]. On the other hand, the induction of the NLPR3 inflammasome is known to increase tau hyperphosphorylation, and in a mouse model, a decrease in Aβ accumulation is observed when the NLRP3 inflammasome is reduced [73,77].

HSV-1-infected neurons in AD individuals were found to have higher concentrations of beta-site APP cleaving enzyme nicastrin, a component of gamma-secretase, which increases Aβ accumulation while decreasing APP [78]. Interestingly, HSV-1 transport is intertwined with APP, utilizing the APP kinesin motor for anterograde movement, which disrupts APP trafficking, leading to impaired localization and proteolysis. This, in turn, enhances amyloidogenic processing and Aβ production [79,80]. The increased Aβ accumulation can also cause hyperexcitability, triggering intracellular calcium signals [81]. On the contrary, a study showed that Aβ is an antiviral agent that can neutralize glycoprotein B in HSV-1 [82].

Two critical enzymes, GSK3β and PKA, which play a role in tau phosphorylation, are upregulated in HSV-1-infected cells, suggesting that HSV-1 may participate in AD progression by enhancing tau hyperphosphorylation [16]. Another study showed that HSV-1-infected neuroblastoma cells increase tau spreading by inducing phosphorylated tau using extracellular vesicles, which function as cell-to-cell communication [83].

### 5.4. Autophagy

Autophagy is the process by which defective proteins or organelles in lysosomes are removed and recycled. In AD patients, autophagy is impaired. In non-AD brains, autophagic vacuoles are quickly cleared by lysosomes, whereas inhibition of late-stage degradation in macroautophagy showed accumulation of LC3-II in neurites that mimic the dystrophic neurites in AD brain [84]. In AD, the final stage of autophagy-lysosomal degradation is usually impaired. Moreover, in AD brains, lysosomal acidification is another factor contributing to autophagy dysregulation, alongside a reduction in the Beclin-1 and VPS34 complex [85,86]. In HSV-1-infected cells, autophagosomes with Aβ also inhibit the fusion between the autophagosome and lysosome [87]. Moreover, HSV-1 ICP34.5 can bind to Beclin-1, which blocks autophagy in neurons and allows the virus to propagate, potentially worsening AD, as Aβ is even more resistant to degradation [17,88].

### 5.5. Metabolic Changes

Metabolic reprogramming from HSV-1 infection is shown to alter neuronal functions, which can worsen the progression of AD. HSV-1 infection shifts host glucose metabolism in the mitochondria from oxidative phosphorylation towards aerobic glycolysis by increasing glucose-6-phosphate and fructose-1,6-bisphophate for the pentose phosphate pathway favoring HSV-1 replication, which disrupts neuron metabolic functions [89]. Additionally, HSV-1 is shown to thicken and shorten the cristae in mitochondria resulting in endoplasmic reticulum stress and an influx of calcium ions, which promotes apoptosis of the mitochondria [90,91]. Specific viral proteins further contribute to mitochondrial dysregulation: UL16 enhances mitochondrial activity by binding to host adenosine nucleoside transporter 2 (ANT2) which increases ATP production, while ICP34.5 binds mitochondrial factors and alters their various proteins such as KEAP1 and PGAM5, though the exact mechanism remains unknown [92,93]. In neurons, HSV-1 also disrupts mitochondrial dynamics, including fission, fusion, and motility, leading to impaired energy distribution and elevated oxidative stress [94,95]. These alterations reduce ATP availability, increase reactive oxygen species, and compromise protein homeostasis, ultimately creating an environment that promotes amyloid accumulation, tau pathology, and neuronal dysfunction in AD.

### 5.6. Small Non-Coding RNAs (sncRNAs) and Their Potential Roles in AD

Small non-coding RNAs (sncRNAs) are less than 200 nucleotides in length. Although they do not encode proteins, they function as important regulators of gene expression. Among sncRNAs, microRNAs have been most extensively studied in the context of AD, where they are known to dysregulate multiple molecular pathways and contribute to disease progression [96]. In contrast, the roles of other sncRNA classes in AD remain at an early stage of investigation. Transfer RNAs (tRNAs), best known for delivering amino acids to codons during protein synthesis, can also give rise to tRNA-derived fragments (tRFs) through enzymatic cleavage of precursor or mature tRNAs. These tRFs, typically 15–30 nucleotides in length, have recently emerged as a novel sncRNA family with regulatory functions in stress responses, human diseases, and viral infections [97,98].

One of our recent findings has demonstrated that tRFs derived from the 5′-end of a limited set of mature tRNA (tRF5) are significantly enhanced by AD in the human hippocampus [99]. The study showed that tRF5s from tRNA ProAGG (tRF5-ProAGG), tRNA GlyGCC (tRF5-GlyGCC), GlyCCC-2 (tRF5-GlyCCC2), and tRNA GluCTC (tRF5-GluCTC) were increased in AD patients [99]. Among those impacted tRFs, tRF5-ProAGG correlates with disease severity and is also important for the expression of genes essential for neuronal functions [99,100].

The mechanisms by which specific tRNAs are selected to produce tRFs are not known. The modifications of tRNAs and those associated with being prone to be cleaved by ribonucleases, such as angiogenin (ANG) and ElaC ribonuclease 2 (ELAC2), are suggested to be essential for tRNA cleavage [101,102,103,104,105]. Recent publications on AD patients with amyloid aggregates have shown reduced expression of tRNA-modifying enzymes, including Elongator acetyltransferase complex subunit 3 (ELP3) and NOP2/Sun RNA methyltransferase 2 (NSUN2), in the hippocampus [80,87]. Enhanced tRF expression also supports the importance of tRNA modifications in tRF production in AD.

Similarly, according to our unpublished observations, the tRF profile can be significantly altered following neurotropic viral infections. For example, we found that HSV-1-infected neurons produce tRF5-GlyCCC2 (manuscript in preparation). Whether commonly affected tRFs contribute to HSV-1-involved AD development is an interesting research topic for us currently. We are also interested in other HSV-1-impacted tRFs and their roles in controlling HSV-1 replication and inducing host inflammatory responses.

In addition to tRFs, microRNAs also play a crucial role in HSV-1 replication. For example, miR-H2-3p is known to reduce ICP0, promoting virus latency, whereas miR-H6 reduces ICP4, which is required for the expression of most HSV-1 genes during productive infection, thereby also contributing to the establishment of HSV-1 latency [106].

### 5.7. AD Patients Are More Permissive to HSV-1

As people age, it is natural for the immune system to decline gradually, which can favor HSV-1 penetration of the blood–brain barrier (BBB). The likelihood of HSV-1 infection in the non-AD and AD brain is worth comparing. So is the mechanism that drives HSV-1 entry into the BBB. It has been shown that the APOE-epsilon 4 allele frequency was significantly higher in patients positive for HSV-1 in the brain than in the HSV-1-negative AD group, the HSV-1-positive non-AD group, or the HSV-1-negative non-AD group (52.8% vs. 10.0%, 3.6%, and 6.3%, respectively), supporting that AD patients are more susceptible to HSV-1 [107]. Comparing HSV-1 infection/replication in CNS cells derived from AD individuals with/without a genetically inherited risk factor or healthy individuals might be further helpful to conclude the impact of AD on the HSV-1 susceptibility in the brain.

In summary, based on current research, HSV-1 is associated with a higher risk of developing AD, in addition to the genetic and environmental factors [108,109]. Moreover, the question of whether AD patients are more likely to acquire recurrent HSV-1 infection in the brain provides insights into future research on the physiological aspect. In a recent study, degeneration of pericytes and the endothelium of the AD BBB was found to be independent of aging. Various enzymes from the coagulation cascade, such as fibrinogen, along with IgG and hemosiderin, were significantly higher in the AD BBB [110].

## 6. Models to Examine HSV-1 in Dementia

Studying brain diseases in humans is challenging due to the limited availability of tissue samples, which are typically only accessible postmortem. To overcome this limitation, various models have been developed to study human brain-related conditions. In particular, several experimental models have been employed to investigate the role of HSV-1 latency and reactivation in the development of AD, the focus of this review. In this section, we will outline the models currently available, highlighting their respective strengths and limitations. The key advantages and limitations of these experimental models are summarized in Table 1.

### 6.1. In Vitro Models (Cell Culture Systems)

Among the available approaches, in vitro models offer a practical and scalable alternative. These include neurons cultured in monolayers, or co-cultured with other physiologically relevant cells, either in monolayers or in trans-well models, as well as more complex 3D organoid models. These in vitro platforms provide researchers with opportunities to overcome the limitations of postmortem tissue, generate large quantities of human-relevant samples, and facilitate high-throughput screening and detailed mechanistic studies.

#### 6.1.1. Cells Cultured in a Monolayer (2D Cell Culture)

Many 2D cell culture models have been developed to study HSV-1 infection, including the B103 murine neuronal cell line, Lund human mesencephalic (LUHMES) cells, Neuro-2A (N2A) and C1300 mouse neuroblastoma cell lines, the rat pheochromocytoma PC12 cell line, the human neural stem cell line ReNcell VM, the human neuroblastoma cell line SH-SY5Y, the human dorsal root ganglion-like cell line HD10.6, and neurons differentiated from human induced pluripotent stem cells (iPSCs). These in vitro systems have been widely used to investigate the potential link between HSV-1 latency and AD [111,112,113,114,115,116,117].

Among these models, several have been employed explicitly in AD-related studies. In SH-SY5Y cells, HSV-1 infection led to increased production and accumulation of Aβ peptides [118,119]. N2A cells have been utilized to investigate the molecular mechanisms underlying miRNA-mediated HSV-1 latency. This study revealed that the neuronal miRNA miR-9 plays a key role in silencing HSV-1 through epigenetic regulation [120].

Additionally, the latency-associated transcript (LAT) of HSV-1 has been shown to protect neurons from granzyme B-mediated cytotoxicity by CD8+ T cells, suggesting a viral strategy to preserve neuronal hosts during latency [121]. Human iPSCs, which can differentiate into neurons, astrocytes, and microglia, provide a valuable platform for modeling cell-type-specific responses to HSV-1. In one study, researchers utilized a co-culture system of iPSC-derived microglia and neurons to show that HSV-1 infection decreased expression of triggering receptor expressed on myeloid cells 2 (TREM2) in microglia to reduce inflammation and suppress phagocytosis of HSV-1-infected neurons, as TREM2 is important for virus-induced *IFNβ* induction through the DNA-sensing cGAS-STING pathway in microglia and for phagocytosis of HSV-1-infected neurons [122].

The transwell system has been used to model the mechanism by which infected immune cells carry HSV-1 across barriers. For example, infected cells can be placed on one side of an endothelial transwell to see if they can pass through and spread the virus to the other side. Such studies will help elucidate whether HSV-1 primarily crosses the BBB by infecting and damaging endothelial cells. The PC12 cell line was used as a transwell in vitro model for HSV-1 infection because it mimics viral latency [123]. Additionally, PC12 cells are commonly used as a model for studying Parkinson’s disease. Parkinson-like conditions can be induced in these cells using 6-hydroxydopamine (6-OHDA) [124].

#### 6.1.2. Three-Dimensional Cultures and Organoids

Three-dimensional (3D) models, such as brain organoids, have emerged as powerful tools for studying neurological diseases. Brain organoids are typically derived from human induced pluripotent stem cells (iPSCs) and contain multiple cell types, including neurons, astrocytes, and oligodendrocyte precursor cells [125]. In some protocols, microglia can also be incorporated [126]. These features enable the investigation of complex neurological phenomena, including amyloid plaque formation, intercellular tau propagation, and neural network dysfunction. Compared to traditional models, organoids offer significant advantages due to their human origin and capacity for long-term culture, enabling the study of chronic pathological changes. For example, HSV-1-infected brain organoids can be maintained for several days, allowing the observation of progressive pathological changes, such as the extracellular accumulation of Aβ [127,128]. Studies have shown that HSV-1 infection in brain organoids increases the levels of phosphorylated tau (p-tau) [129]. Additionally, it leads to a reduction in the expression of the HSV-1 ICP27 protein and decreases neuronal cell death by activating the cGAS–STING–TBK1 signaling pathway [112]. Another study demonstrated that brain organoid models exhibit different patterns of Aβ immunoreactivity compared to 2D monolayer neuronal cultures [130]. In that study, Aβ accumulation was observed predominantly in non-infected neighboring cells surrounding HSV-1-infected cells. In contrast, their 2D model showed greater Aβ accumulation within the HSV-1-infected cells themselves [130]. Recently, a study using an organoid model showed that persistent mechanical injury can induce HSV-1 reactivation, which can lead to AD [128].

However, organoids still have limitations. They lack key features of the mature human brain, specifically a functional vasculature and a fully developed immune system. Microglia are often absent unless introduced exogenously, and the cellular composition of organoids more closely resembles that of the developing fetal brain rather than the aged brain. These differences may influence their responses to viral infection and protein aggregation, which are critical in neurodegenerative disease research.

### 6.2. Animal Model

Rodent models of HSV-1 and AD: Animal studies, particularly in mice, allow researchers to examine the association between HSV-1 and AD in the context of a whole organism with an intact immune system, blood–brain barrier, and aging processes. Both acute infection and chronic latent infection models have been used, each providing different insights.

In acute HSV-1 encephalitis models, the virus is administered directly into the central nervous system (e.g., intracerebral or intranasal inoculation) and rapid effects are observed. Such infections can cause severe herpes simplex encephalitis (HSE), and surviving animals often display neuroinflammatory damage. One study showed that HSV-1 infection of the olfactory tract of mice induced changes in amyloid precursor protein (APP), localized Aβ accumulation in the brain, and microglial activation resembling early AD lesions [30,73]. However, these acute models can be extreme. The dose of infection required to penetrate the brain often results in significant neuronal loss and HSE pathology, which does not represent the slow neurodegeneration of AD.

Therefore, researchers have utilized chronic models to mimic the lifelong latent HSV-1 infection observed in humans. In a groundbreaking chronic reactivation model, De Chiara and colleagues established HSV-1 latency in mice, followed by repeated reactivation of the virus to simulate lifelong recurrent infections [43]. In this model, young adult mice established latency in the trigeminal ganglion via peripheral infection and were then subjected to mild heat stress at regular intervals to induce HSV-1 reactivation [131]. Surprisingly, following these mild reactivations, the mice progressively developed AD-like neuropathology, including amyloid accumulation, tau hyperphosphorylation, and neuroinflammation—even without overt encephalitis [43]. This study demonstrated that mild, recurrent HSV-1 infections in vivo can cumulatively produce an AD-like phenotype in wild-type mice, mirroring the lifelong reactivation seen in HSV-1-positive humans.

Other in vivo models have focused on the interaction between HSV-1 and established genetic models of AD. Researchers have infected transgenic AD mice to determine whether HSV-1 modulates disease severity. A recent study assessed the outcome of exposing transgenic 5xFAD mice (carrying human APP/PSEN1 mutations) to HSV-1. They found that HSV-1 infection accelerated AD-like pathology in these mice. Mechanistically, the virus induced a robust microglial response with clusters of microglia surrounding HSV-1-positive neurons, and activated the NLRP3 inflammasome pathway, leading to the secretion of inflammatory cytokines that promote Aβ accumulation. Pharmacological inhibition of NLRP3 reduced amyloid burden and alleviated cognitive decline in infected 5xFAD mice [73].

### 6.3. Microfluidic Model (Brain-on-a-Chip)

Neurodegenerative diseases such as AD are challenging to model in non-human species, as they do not naturally develop these conditions. This presents limitations in studying disease mechanisms using traditional animal models. Although brain organoids have provided new insights, they also have inherent constraints, including limited nutrient diffusion due to the absence of vasculature and difficulties in imaging thick tissue structures. To overcome these challenges, advanced models such as brain-on-a-chip systems have been developed. These microfluidic platforms create a controlled microenvironment, support three-dimensional architecture beyond traditional cultures, and facilitate nutrient exchange [132]. Moreover, they can incorporate features of BBB, making them valuable tools for studying how pathogens, such as HSV-1, cross the BBB and invade the central nervous system. Zhang and colleagues demonstrated that HSV-1 induces the suppression of autophagic flux in microglia using a microfluidic chip known as the human neurovascular unit [133].

### 6.4. Biomarkers for HSV-1-Induced AD

HSV-1 is a human-specific virus, and although it can be experimentally introduced into mice, this model does not fully recapitulate the complexity of HSV-1-associated pathology in humans. Because spontaneous reactivation of HSV-1 rarely or never occurs in mice, identifying reliable biomarkers is essential for improving early diagnosis and timely intervention [134,135]. Early-stage detection allows for more effective treatment strategies before irreversible damage occurs. Traditionally, many biomarkers have been identified using antibody-based detection methods. However, these often rely on species-specific protein recognition, limiting their translational applicability across models. Recently, RNA biomarkers, including tRFs, have emerged as promising non-protein biomarkers for AD, offering potential advantages in sensitivity, given that the signals can be amplified by qPCR [99].

## 7. Conclusions

Over half of the global population is infected with HSV-1. Simultaneously, AD remains a health burden, especially within the aging population [1]. The recurrent reactivation of HSV-1 infection is shown to increase the progression of AD through chronic neuroinflammation, oxidative stress, amyloid-β accumulation, tau hyperphosphorylation, and impaired autophagy. Although many studies have demonstrated an association between HSV-1 and AD, further exploration is needed to determine whether HSV-1 infection is a cause or a consequence of AD degeneration. Because HSV-1 is latent in the trigeminal ganglion and travels to the brain during reactivation, a model that can physiologically mimic human-brain conditions remains a challenge. Thus, future studies should examine possible experimental models in order to determine the causality between HSV-1 and AD.

## Figures and Tables

**Table 1:** Advantages and disadvantages of different models for HSV-1 and AD research.

Model	Advantages	Disadvantages	Applications in AD Research
Monolayer	Simple, reproducible Easy virus infection and readout of molecular pathways	Lacks 3D structure Limited cell–cell interactions	Mechanisms of HSV-1 entry/replication in neurons or glia APP processing and Aβ production after infection
Transwell system	Models barrier permeability (e.g., BBB) Easy to separate apical vs. basolateral compartments	No blood flow or shear stress More complex than monolayer	HSV-1 crossing BBB and infection of brain-side cells Test antiviral/anti-inflammatory drugs on barrier integrity
3D culture system (Brain organoids)	Human origin, complex neural networks Long-term culture Patient-derived possible	Lacks vascular and immune system Expensive, variable Mimic fetal rather than aged brain	HSV-1 infection dynamics in human brain tissue-like environment Induction of AD-like phenotypes (Aβ deposition, tau phosphorylation)
Animal models	Whole organism with intact immune system, BBB, aging Genetic engineering possible	Not natural AD Species differences in HSV-1 latency and reactivation Costly, time-consuming	Study HSV-1 latency/reactivation in the nervous system Correlate chronic HSV-1 infection with AD-like pathology and behavior
Microfluidic models (Brain-on-a-chip)	Dynamic flow and nutrient exchange Can include BBB-like systems Real-time monitoring	Specialized devices and expertise needed Expensive	Track HSV-1 transport across BBB-like structures Monitor inflammatory cascades affecting neurons and astrocytes

## Data Availability

No new data were created or analyzed in this study. Data sharing is not applicable to this article.
